# Perspective of Artificial Intelligence in Disease Diagnosis: A Review of Current and Future Endeavours in the Medical Field

**DOI:** 10.7759/cureus.45684

**Published:** 2023-09-21

**Authors:** Vidhya Rekha Umapathy, Suba Rajinikanth B, Rajkumar Densingh Samuel Raj, Sankalp Yadav, Sithy Athiya Munavarah, Ponsekar Abraham Anandapandian, A Vinita Mary, Karthika Padmavathy, Akshay R

**Affiliations:** 1 Public Health Dentistry, Thai Moogambigai Dental College and Hospital, Dr. MGR Educational and Research Institute, Chennai, IND; 2 Paediatrics, Faculty of Medicine-Sri Lalithambigai Medical College and Hospital, Dr. MGR Educational and Research Institute, Chennai, IND; 3 Traumatology and Orthopaedics, Kursk State Medical University, Kursk, RUS; 4 Medicine, Shri Madan Lal Khurana Chest Clinic, Moti Nagar, New Delhi, IND; 5 Pathology, Sri Lalithambigai Medical College and Hospital, Dr. MGR Educational and Research Institute, Chennai, IND; 6 Prosthodontics, Thai Moogambigai Dental College and Hospital, Dr. MGR Educational and Research Institute, Chennai, IND; 7 Computer Science and Engineering, School of Computer Science and Engineering, Vellore Institute of Technology, Vellore, IND

**Keywords:** mobile health, expert systems, deep learning (dl), ai and machine learning, medical diagnosis, artificial intelligence, ai

## Abstract

Artificial intelligence (AI) has demonstrated significant promise for the present and future diagnosis of diseases. At the moment, AI-powered diagnostic technologies can help physicians decipher medical pictures like X-rays, magnetic resonance imaging, and computed tomography scans, resulting in quicker and more precise diagnoses. In order to make a prospective diagnosis, AI algorithms may also examine patient information, symptoms, and medical background. The application of AI in disease diagnosis is anticipated to grow as the field develops. In the future, AI may be used to find patterns in enormous volumes of medical data, aiding in disease prediction and prevention before symptoms appear. Additionally, by combining genetic data, lifestyle data, and environmental variables, AI may help in the diagnosis of complicated diseases. It is crucial to remember that while AI can be a powerful tool, it cannot take the place of qualified medical personnel. Instead, AI ought to support and improve diagnostic procedures, enhancing patient care and healthcare results. Future research and the use of AI for disease diagnosis must take ethical issues, data protection, and ongoing model validation into account.

## Introduction and background

An international focus on enhancing the effectiveness of healthcare services using information technology has resulted from an aging patient population and a scarcity of medical personnel. Several health-related sectors heavily rely on artificial intelligence (AI) techniques such as machine learning (ML) and deep learning (DL). These sectors encompass the development of new healthcare systems, the management of patient information and records, and the treatment of various disorders [1,2]. AI is an area of algorithm-based software that enables robots to use information to solve problems. It may replicate human thought processes and intellectual activities. AI is frequently employed in the medical industry and can help with the creation of new treatments in the information age. AI may identify precise medicines for complicated diseases, improve the course of care for patients with chronic diseases, and lower medical mistakes [3]. AI now comes in two main categories. Expert systems are the first category. An expert system is a computer program that can make better decisions than human decision-makers while generating forecasts under close monitoring. It is made up of an inference engine and a knowledge base, two interconnected subsystems. The knowledge base encompasses the collection of accumulated experiences, while the inference engine, which serves as a reasoning system, is capable of accessing the current state of the knowledge base and incorporating further information into it. Expert systems can speed up prototyping, facilitate maintenance, and produce more important information for the system [4]. Expert systems have several limitations, nevertheless, in terms of performance and knowledge accumulation. Long used in medical practice, computer-assisted methods have recently produced very modest advancements. ML is the second kind. This is the foundation of AI and a key strategy for giving computers intelligence. For training, ML needs a huge amount of data. Their performance during the procedure is systematically improved by this. Parameter screening is one of the key objectives of ML. The potential enhancement of AI efficiency by parameter reduction must be considered with the potential trade-off of reduced accuracy. Excessive parameters might introduce errors in inputs and computations, compromising the overall accuracy of the AI system. However one of the main goals of AI is to surpass humans by learning on the job in difficult sectors without any prior training.

## Review

AI technologies, including support vector machines, classification trees, and artificial neural networks, have been essential in facilitating the clinical diagnosis of several acute and chronic illnesses. Notably, these systems have demonstrated efficacy in diagnosing conditions such as acute appendicitis [5] and Alzheimer's disease [6]. The ability of integrative AI to detect cancerous cells is greatly enhanced by using many algorithms as opposed to a single algorithm, leading to improved diagnostic accuracy [7]. The development of several AI approaches helps to forecast the recurrence of breast cancer [8]. Instead of doctors, at-home AI systems might possibly monitor patients with insulin irregularities and swallowing issues [9]. Healthcare is only one of several industries where AI has made major advancements in recent years. The potential of AI in disease diagnosis has generated a lot of interest and study. This review will cover the advantages, drawbacks, and ethical issues surrounding AI's current role in disease detection as well as its potential in the future. The influence of AI on medical diagnostics is anticipated to expand dramatically as healthcare systems adopt digital technology and AI algorithms continue to advance.

**Figure 1:**
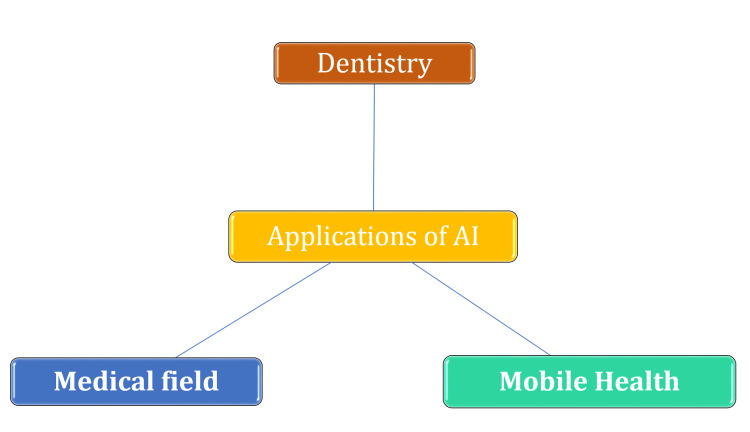
Implication of AI in various fields AI: artificial intelligence

Medical professionals frequently face novel challenges as a result of evolving responsibilities and frequent interruptions within the healthcare system, which is characterized by its dynamic and ever-changing nature [10-12]. Medical workers often encounter new obstacles due to expanding responsibilities and frequent disruptions within the healthcare system, which is known for its dynamic and constantly changing nature [13]. Because the disease can develop over time and patient dynamics might alter [14,15], medical specialists' available time is often constrained [16,17]. Additionally, diagnostics can be a very difficult procedure [18,19]. The concept of AI typically refers to the ability of a machine to engage in cognitive processes that are commonly associated with human intelligence, including but not limited to perception, reasoning, learning, environmental interaction, problem-solving, decision-making, and potentially even creative expression [20]. The buzz and enthusiasm surrounding AI continue. The fervor and excitement surrounding artificial intelligence persist [21-23], and both researchers and practitioners place equal emphasis on this technology from a variety of angles [24-27]. AI encompasses a wide variety of scientific fields, including robotics and natural language processing, and is typically linked with human-like behavior [28,29]. While ML is being used to generate these practical applications, they are now focused on a single job [30] and include healthcare and illness diagnoses. In order to make predictions, algorithms use medical data [28] and continually learn and improve over time by processing fresh and updated data. [31]. Algorithms acquire knowledge via diverse inputs and sources of information, as well as during extensive periods of experiential learning [31]. As a result, AI-enabled computers can digest more information than people can [32], perhaps surpassing humans for some medical activities. The use of AI to help medical practitioners in the diagnosing process might be extremely beneficial for the healthcare industry and patients' overall health. With AI in existing technical infrastructure, the process of identifying pertinent medical information from several sources that aligns with the patient's requirements and treatment plan is expedited (Figure 1) [33-36].

Role of AI in the medical field

Medical diagnostics refers to the systematic evaluation of medical problems or diseases through the examination and interpretation of symptoms, medical history, and test results. The primary objective of medical diagnostics is to ascertain the underlying etiology of a medical condition and provide a precise diagnosis, thereby facilitating the administration of suitable therapeutic interventions. The diagnostic process may involve the utilization of several techniques, such as imaging modalities including X-rays, MRIs, and CT scans, as well as blood tests and biopsy procedures. The results of these tests assist healthcare providers in determining the optimal treatment approach for their patients. Medical diagnostics serve multiple purposes, including monitoring the evolution of a condition, assessing the effectiveness of therapy, detecting potential health concerns at an early stage, and facilitating the identification of medical disorders. One potential optimal intelligent approach that could potentially enhance diagnostic outcomes, drawing upon a range of findings from images, signals, textual representations, and other sources, is the use of multimodal patient data diversity. The utilization of multimodal data in healthcare practice enables healthcare providers to enhance their ability to effectively treat and manage chronic diseases by monitoring the progression of a condition over an extended period. Healthcare professionals utilizing Explainable XAI can identify possible health issues sooner, before they become significant and perhaps fatal, by leveraging multimodal medical data [37]. Additionally, AI-powered Clinical Decision Support Systems (CDSSs) might offer in-the-moment help and guidance to make better judgments on patient care. Healthcare practitioners may concentrate on more complicated patient care by using XAI tools to automate common procedures.

There is a good chance that OpenAI will continue to expand and improve in the future of AI-based medical diagnostics. Quantum AI (QAI), a more sophisticated AI technology, is being brought into the research community in order to accelerate the traditional training process and offer quick diagnostic models [38]. Due to quantum computers' much-increased processing capability over conventional computers, real-time analysis of enormous volumes of medical data using quantum AI algorithms may result in diagnoses that are more precise and effective. Decision-making procedures in medical diagnostics, such as selecting the best course of therapy for a patient based on their medical history and other characteristics, can be optimized using quantum optimization algorithms. Another idea is GAI, or generic AI, which is employed by a variety of initiatives and businesses, including DeepQA from OpenAI, Watson from IBM, and DeepMind from Google. The primary aim of utilizing AI in medical diagnostics is to enhance the accuracy, promptness, and efficacy of diagnosing medical conditions. Additionally, it aims to provide essential information and support to healthcare practitioners in the process of diagnosing and caring for patients. The implementation of general AI in medical diagnostics has the potential to significantly transform the field of medicine. By using AI algorithms, this approach enables the analysis of vast quantities of medical data, facilitating the identification of patterns and correlations. This intervention is expected to improve patient outcomes and contribute to the development of a more efficient and effective healthcare system. The utilization of AI in the field of medical diagnostics is now in its nascent stages of advancement, necessitating the resolution of various technological, legal, and ethical concerns prior to its comprehensive implementation. In order for AI algorithms to be effective, they need a lot of high-quality labeled data, which can be difficult in the medical area since data is frequently fragmented, partial, unlabeled, or missing. In addition, various businesses and organizations frequently create AI-based medical diagnostic tools; hence, interoperability standards and protocols are required to guarantee that these products can function properly together. Personalized treatment plans may be created using AI-based approaches that examine a patient's medical history, genetics, and other aspects; this development is anticipated to continue in the future. Nevertheless, due to the existing knowledge gaps in AI-based medical diagnostics, it is highly recommended that researchers conduct additional investigations to enhance the accuracy of the ultimate prognostications and expedite the process of knowledge acquisition.

AI and dentistry

One of the nine dental disciplines, orthodontics focuses on diagnosing malocclusions with the ultimate goal of preventing and treating them. The craniofacial skeleton is its major focus, and the dentoalveolar region is modified more than other areas. The efficacy of orthodontic treatment is believed to be contingent upon a precise diagnosis and comprehensive treatment planning, necessitating a high level of meticulousness from orthodontic practitioners in both domains. Cephalometric radiographs are often regarded as a valuable tool in orthodontic diagnosis due to their ability to assess abnormalities pertaining to the dental and craniofacial bones. The process of diagnosing orthodontic conditions primarily relies on several key factors, including the patient's dental and medical history, a thorough clinical examination, the use of study models, and the analysis of cephalometric radiographs. The field of dentistry has experienced a substantial evolution in recent times. Recent advancements have facilitated the development of technology that emulates the cognitive processes of the human brain. These mostly rely on AI technology, which has contributed significantly. These automated technologies have been effective in aiding doctors in treatment planning and diagnostic prediction [39]. These AI-based systems have been utilized as tools to help orthodontists provide a consistent level of patient care and increase the likelihood that the objectives will be met [40]. The expert can use AI technology to make better healthcare decisions [41]. The definition of AI technology is the simulation of human intelligence by a computer that is capable of logical thought and action [42,43].

AI has been utilized in the field of clinical dentistry for many purposes, such as disease prevention, diagnosis, treatment planning, prognosis, and enhancing environmental sustainability [44,45]. A novel AI-driven voice-activated software application, known as Dragon Ambient Experience (DAX) Express, has been introduced by Nuance Communications, Inc., based in Burlington, MA, USA. This program aims to facilitate real-time transcription of medical records during patient consultations by leveraging natural language processing techniques. The medical field of dentistry is one with significant levels of occupational stress and burnout [46]. Using DAX in clinical dentistry might help dentists capture medical records verbatim.

Additionally, DAX will automate standard notes and add them to the patient record in a matter of hours. Consequently, the process of examining the automated summaries of the patient's visit and the treatment plan is straightforward for the dentist. Time might be saved, and job stress and burnout could be reduced. Typically, a wide variety of medical malpractice lawsuits occur through third-party-evaluated medical data. According to reports, around 35.7% of dental conflicts in Taiwan result in criminal convictions. The utilization of AI in the creation of medical records and informed consent documents will result in the standardization of these documents, ensuring accuracy and readability. This standardization is made possible with the assistance of DAX technology. As a result, it could help reduce or stop medical disagreements. First, DAX will aid dentists in reducing job fatigue. Second, DAX could enhance informed consent and enhance medical record-keeping. Finally, from a long-term perspective, DAX would reduce medical disagreements. Additionally, it could be possible to calculate the unexpected aspects of medical malpractice analyses. As a result, the insurance company may use the data to evaluate and create the appropriate insurance policies. Perhaps both medical treatment and the absence of medical disputes would result in a win-win situation. However, in order to use AI in clinical dentistry, patient autonomy, informed consent, and morality have to be modified [47]. These crucial ethical problems still require more research.

Mobile health

Initially, information technology systems primarily served the purpose of gathering patient data. However, as technological advancements have rapidly progressed, the healthcare industry has been able to leverage data analytics and machine learning techniques [48]. The field of digital health care and preventative medicine has witnessed a notable surge in research due to the advancements in AI techniques and the rapid integration of medical internet of things (IoT) devices [49]. Such studies concentrate on mobile health (mHealth) technologies that are employed to keep track of life-threatening conditions including asthma, diabetes, and sleep apnea and to assure the safety and well-being of patients [50]. A crucial area of the healthcare IT market that has experienced recent significant growth is mHealth [51]. Wearable technology adoption, the proliferation of mobile sensors, and the exponential rise in IoT device numbers overall have all contributed to this trend [52-54]. Wearing health devices outside of hospitals for remote in-home care has expanded concurrently with the rise in IoT device use. As a result, there is now more research being done and more money being invested in mHealth. Researchers have emphasized the significance of mHealth in difficult situations like the current pandemic to enable the provision of remote medical facilities [55].

Since COVID-19, the use of mobile health has significantly increased, according to a recent study [56]. These capabilities are intensive in guaranteeing the preservation of social distance and better cleanliness. The degree of AI available to researchers has significantly improved along with the growth in mHealth research. While simultaneously protecting patient privacy and guaranteeing a high level of data security, these advancements provide more accurate insights and outcomes than classical ML. These more recent methods that guarantee data security and privacy include DL and federated learning (FL) [57]. Numerous systematic literature reviews highlight the significance of AI in this field, which has been effectively integrated with the healthcare industry [58-60]. Recently, the amount of research into AI methods in the mHealth industry has significantly expanded. This can be due to the COVID-19 pandemic's quick evolution and adoption of telemedicine. Researchers have recorded disease progression [61] using mHealth sensors, showing how an ailment spreads or develops in a patient over time. The early detection and treatment of chronic diseases, as well as the management of symptoms that have up until now eluded standard patient monitoring in hospitals and assisted living facilities, can greatly benefit from these insights.

A new study field that examines the fusion of these two research streams has emerged as a result of the convergence of AI and mHealth. It is referred to as AIM (AI-powered mHealth). Numerous advantages of applying AI techniques to mHealth scenarios include automatic chronic disease occurrence detection, real-time suicide prediction and intervention [62], facilitating emergency response [63], enabling patient rehabilitation, providing noninvasive care [64,65], and avoiding medical errors. In the USA, medical mistakes that might have been avoided are a major cause of mortality. Utilizing real-time information from wearable health sensors, clinical decision-making technologies can greatly minimize it [66]. AIM can also make it simple for at-risk minority communities to get high-quality medical treatment even when they lack access to medical facilities [67]. Earlier studies concentrating on the use of AI in the healthcare industry found that certain implementation barriers exist that prohibit the healthcare industry from being extensively automated [68]. However, there has been a huge increase in both AIM research and practice as a result of the development of DL and AI approaches. Both the use of AI and mHealth have made considerable strides over the past few years. In this sense, a number of recent studies that aim to explain and apply the therapeutic application of AI in mHealth contexts (AI + mHealth) [69,70] have a common framework.

Another crucial point to keep in mind is how mHealth and telehealth have developed into reputable methods for giving patients noncritical care, which is essential in the midst of the present epidemic. This review examines current advancements in the AIM field, and based on our results, we provide some useful suggestions for ongoing study. First, AIM might enable an even wider growth and supply of mHealth services by using the most current advancements in AI approaches, FL and XAI, as shown by its considerable usage during the epidemic [56]. Second, an agreement about the guidelines governing the use of wearable technology in medical facilities might be developed as a result of the greater acceptance of such services. Hospitals now use exclusive mobile health equipment that forbids data sharing, even if it is necessary for urgent research. Given the expanding significance of telehealth and remote health services during the COVID-19 pandemic, we come to the conclusion that it is crucial to encourage wider use of patient privacy protection, smart wearable health device adoption, and remote health monitoring devices. This is due to the fact that our research demonstrates that mHealth does, in fact, significantly influence how people attend healthcare facilities in tumultuous situations like a pandemic. Some current AI methods that can hasten acceptance and enable quicker deployment throughout hospitals, medical institutions, and users at large are explored in this context. The smart healthcare revolution can be successfully fueled by ML techniques like DL, FL, and transfer learning to secure patients' privacy.

The creation of effective algorithms is a crucial result of using DL in the mHealth analytics sector, as we see from the analysis of the publications included in this study. It gets harder for researchers working in this field to obtain user data since it is scattered among several hospitals and medical institutes in isolated silos or islands. Furthermore, lacking individualized information about each person, it becomes challenging to generalize an ML model's performance for a wide population. Since user data never leaves an organization, it guarantees data privacy [71]. Additionally, model knowledge gained from one piece of data may be applied to forecast results from a different collection of data. The use of FL models ensures that data are static and located at the source, ensuring privacy. Under such models, stochastic gradient descent is represented solely numerically and is the only form of information transmission.

The Defense Advanced Research Projects Agency's XAI program aims to encourage the creation of AI systems whose models can be used, understood, and trusted by end users [72]. XAI is essential for the development of AIM integration since it may improve the acceptance and comprehension of ML methods and models in the healthcare industry. According to several studies on the technological acceptance model [73], we may anticipate a rise in the adoption rate of AIM in the healthcare sector as our understanding of AI models grows. This concept states that people's intentions and behaviors to participate in and utilize the aforementioned technology rise as its use becomes easier. In this situation, the broad use of AI models would greatly boost hospitals' efficacy as they become easier to use and implement. In order to get over the limitations that limited information and comprehension impose on the usage of AIM, one must go inside the opaque nature of AI. The demonstration of the use of XAI by Gordon et al. [74] approaches the analysis of medical data for real-time clinical decision assistance in surgical and operating settings in hospitals.

These models can aid surgical teams in the analysis, foreseeing, comprehension, and prevention of unfavorable intraoperative occurrences. A different study by Payrovnaziri et al. examined the potential of XAI to mimic actual electronic health record data [75]. They conclude that XAI has not been effectively researched and applied in medicine after pointing out various gaps in the literature. They admit that there are a number of possibilities where the adoption and use of XAI might considerably improve mHealth. Both research and practice stand to benefit significantly from these. The recentness of these studies highlights the significance of AIM in the healthcare industry and offers a direction for further study in this crucial area. This work has some limitations, much like the majority of scoping evaluations. First, we solely took into account research in the three fields of biomedical technology, information science, and artificial intelligence. Second, although it is a developing area of medical study, we did not take into account the societal implications of AIM technology in our article. We will do our best to overcome these constraints in our next efforts. Such work comprised articles that were included in a specific database, such as the ACM Digital Library, and published in specialized ML and AI conference proceedings. However, the databases we chose are thorough resources for contemporary AIM research and contain the most recent peer-reviewed research literature in the three streams. Assuring cooperation and data exchange across various medical organizations is a way to address this issue. These cooperative efforts will guarantee that hospitals, doctors, and other healthcare professionals use AI tools more effectively. Additionally, academics are developing new and sophisticated AI approaches at a rapid rate, such as FL and XAI, and their eventual implementation in real-world settings will probably have life-saving effects in the future.

## Conclusions

In the current state of healthcare, the use of AI in disease diagnosis has already shown encouraging outcomes. As technology develops, AI's potential for improving the detection of uncommon diseases, providing access to healthcare through telemedicine, and practicing predictive and preventative medicine will only grow. While AI has a lot to offer in terms of disease detection, issues with data quality, interpretability, and ethics need to be resolved if deployment is to be fair and responsible. AI has the potential to be a powerful partner in the battle against disease, enhancing medical knowledge and patient outcomes with the right strategy.
